# The cellular adaptor GULP1 interacts with ATG14 to potentiate autophagy and APP processing

**DOI:** 10.1007/s00018-024-05351-8

**Published:** 2024-07-30

**Authors:** Dennis Dik-Long Chau, Zhicheng Yu, Wai Wa Ray Chan, Zhai Yuqi, Raymond Chuen Chung Chang, Jacky Chi Ki Ngo, Ho Yin Edwin Chan, Kwok-Fai Lau

**Affiliations:** 1 School of Life Sciences, Faculty of Science, The Chinese University of Hong Kong, Hong Kong SAR, China; 2 Laboratory of Neurodegenerative Diseases, School of Biomedical Sciences, LKS Faculty of Medicine, and State Key Laboratory of Brain and Cognitive Sciences, The University of Hong Kong, Hong Kong SAR, China; 3 Laboratory of Drosophila Research, The Chinese University of Hong Kong, Hong Kong SAR, China

**Keywords:** Amyloid precursor protein, Autophagy-related 14, LC3, Macroautophagy, GULP1

## Abstract

Autophagy is a highly conserved catabolic mechanism by which unnecessary or dysfunctional cellular components are removed. The dysregulation of autophagy has been implicated in various neurodegenerative diseases, including Alzheimer’s disease (AD). Understanding the molecular mechanism(s)/molecules that influence autophagy may provide important insights into developing therapeutic strategies against AD and other neurodegenerative disorders. Engulfment adaptor phosphotyrosine-binding domain-containing protein 1 (GULP1) is an adaptor that interacts with amyloid precursor protein (APP) to promote amyloid-β peptide production via an unidentified mechanism. Emerging evidence suggests that GULP1 has a role in autophagy. Here, we show that GULP1 is involved in autophagy through an interaction with autophagy-related 14 (ATG14), which is a regulator of autophagosome formation. GULP1 potentiated the stimulatory effect of ATG14 on autophagy by modulating class III phosphatidylinositol 3-kinase complex 1 (PI3KC3-C1) activity. The effect of GULP1 is attenuated by a GULP1 mutation (GULP1m) that disrupts the GULP1–ATG14 interaction. Conversely, PI3KC3-C1 activity is enhanced in cells expressing APP but not in those expressing an APP mutant that does not bind GULP1, which suggests a role of GULP1–APP in regulating PI3KC3-C1 activity. Notably, GULP1 facilitates the targeting of ATG14 to the endoplasmic reticulum (ER). Moreover, the levels of both ATG14 and APP are elevated in the autophagic vacuoles (AVs) of cells expressing GULP1, but not in those expressing GULP1m. APP processing is markedly enhanced in cells co-expressing GULP1 and ATG14. Hence, GULP1 alters APP processing by promoting the entry of APP into AVs. In summary, we unveil a novel role of GULP1 in enhancing the targeting of ATG14 to the ER to stimulate autophagy and, consequently, APP processing.

**Supplementary Information:**

The online version contains supplementary material available at 10.1007/s00018-024-05351-8.

## Introduction

Macroautophagy, hereafter referred to as autophagy, is a highly conserved metabolic mechanism by which unnecessary or dysfunctional cellular components are removed via sequestration in double-membrane vesicles known as autophagosomes. These autophagosomes then fuse with lysosomes to form autolysosomes, which leads to the degradation of the sequestered material and the eventual recycling of the resulting macromolecules. Autophagy is also thought to play roles in the metabolism of disease-related proteins, including amyloid precursor protein (APP), which is associated with Alzheimer’s disease (AD). The aggregation of amyloid-β peptide (Aβ), which is formed by the cleavage of APP by β- and γ-secretases, is a key pathological process in AD. Aβ is reported to be produced in autophagosomes [1], and oxidative stress-induced autophagy enhances Aβ production [2]. Furthermore, reductions in Aβ secretion and the plaque burden have been observed in an autophagy-deficient mouse model [3]. However, treatment with rapamycin, an autophagy inducer, has been shown to reduce intracellular Aβ levels and plaque loads in mouse models of AD [4, 5]. There is increasing evidence that autophagy plays dual roles in neurodegeneration by (i) stimulating the clearance of abnormal proteins and (ii) perturbing global proteolysis, thus promoting protein aggregation. These findings highlight the complex roles of autophagy in both Aβ production and clearance [6, 7].

Engulfment adaptor phosphotyrosine-binding (PTB) domain-containing protein 1 (GULP1) and its *Caenorhabditis elegans* homolog CED-6 are cellular adaptors that regulate apoptotic cell corpse engulfment through their interactions with various transmembrane receptors [8–11]. Increasing evidence suggests that GULP1/CED-6 participates in autophagy. CED-6 has been reported to play a role in the recruitment of autophagosomes to phagosomes [12]. Moreover, GULP1 regulates Nrf2-KEAP1 signaling, which is associated with autophagy [13–15]. GULP1 has also been reported to interact with clathrin assembly lymphoid–myeloid leukemia (PICALM), a molecule that has been implicated in modulating autophagic activity [16]. An increase in 17β-estradiol levels is observed in GULP1-knockout (KO) mice [17]. Despite its inconsistent effects, 17β-estradiol has been reported to modulate autophagy [18]. However, the exact mechanism by which GULP1/CED-6 participates in autophagy remains elusive. Of note, GULP1 has been shown to alter Aβ generation [19–21]. Hence, the aforementioned findings suggest an association between GULP1, APP processing and autophagy.

Notably, autophagy is a highly regulated process that involves the formation of various autophagic protein complexes at different stages. For example, class III phosphatidylinositol (PI)3-kinase complex 1 (PI3KC3-C1) is essential for autophagosome initiation. It comprises four major components, namely the lipid kinase catalytic Vps34 subunit, scaffold Vps15, regulatory ATG14 and Beclin1 subunits [22]. It has been reported that some molecules modulate autophagy through interactions with components of the PI3KC3-C1 complex [23, 24]. As GULP1 is an adaptor protein and has been implicated in autophagy, we hypothesized that GULP1 participates in autophagy through an interaction with an autophagy-related protein. Here, we show that GULP1 potentiates autophagy by interacting with ATG14.

## Materials and methods

### Plasmids

The mammalian expression constructs for APP695, HA-tagged full-length GULP1 and untagged GULP1 were as described [19, 20, 25]. Bacterial GST-APPc and 6X-His-GULP1 PTB (residues 1 to 168) were as described [19, 20, 26]. Flag-tagged full-length human ATG14 was obtained from Genscript. Point mutations of GULP1 and ATG14 were generated by QuikChange II site-directed mutagenesis kit (Agilent). APP-GAL4 construct consisting of human APP695 followed by the entire GAL4 transcription factor (pRc-CMV-APP695) was as described [27]. UAS-dependent firefly luciferase reporter pFR-Luc and transfection efficiency vector Renilla luciferase phRL-TK plasmids were obtained from Stratagene and Promega, respectively.

### Antibodies

Rat anti-GULP1 and rabbit anti-APP were as described [19]. Mouse anti-APP (22C11) was purchased from Merck. Mouse anti-HA (12CA5) was purchased from Roche. Mouse anti-FLAG (M2) was purchased from Sigma. Goat anti-GULP1 (P19) and mouse anti-α-tubulin (DM1A), Beclin1 (E-8), Vps15 (JK-13) and Vps34 (F-11) were purchased from Santa Cruz Biotechnology. Rabbit anti-ATG14 and rabbit anti-LC3B were purchased from Proteintech. Mouse anti-GFP (JL-8) was purchased from Clontech. Rat anti-ATG14 and anti-Beclin1 (Bec-R3) were generated by immunizing rats with recombinant ATG14^247−492^ and Beclin1^141−270^, respectively.

### Cell culture and transfection

Chinese hamster ovary (CHO) and human embryonic kidney 293 (HEK293) were cultured as described previously [19, 20, 26] and transfected either by Endofectin Max (Genecopoeia), X-tremeGENE HP (Roche) or Polyethylenimine (Polysicences) following manufacturer’s instruction. siRNA knockdown (KD) was performed with RNAiMax (Invitrogen) according to manufacturer’s instruction. GULP1 and ATG14 ON-TARGETplus siRNAs were purchased from Horizon Discovery. GULP1 knockout HEK293 cells were generated by CRISPR-Cas9 system as described previously [20] using the following guide oligonucleotides, sense 5′–CACCGAAGTTGTGAGAGATGCTGTA–3′; anti-sense 5′–AAACTACAGCATCTCTCACAACTTC–3′, which target the exon 5 of human GULP1 gene.

### Measurement of autophagic flux

GFP-LC3 cleavage, LC3 turnover, p62 turnover assays were performed as previously described to monitor the autophagic flux in cells [28–30]. For the GFP-LC3 cleavage assays, HEK293 cells were transfected GFP-LC3 construct together with either DNA plasmids or siRNAs. The transfected cells were treated with 10 μM CQ for 24 h before harvest. For the LC3 turnover assay, HEK293 cells with stable expression of GULP1 or GULP1 K66A/K69A mutant were treated with 150 nM Baf A1 for 1 h before harvest. The same assay was also performed in GULP1-KO HEK293 cells. For the p62 turnover assay, GULP1-KO HEK293 cells were treated with 150 nM Baf A1 for 1 h. Levels of free GFP, endogenous LC3-II, p62 and α-Tubulin in the cell lysates were analysed by immunoblotting. The relative protein levels (free GFP/α-Tubulin; LC3-II/α-Tubulin; p62/α-Tubulin) indicate the autophagic flux in the cultured cells.

### Immunofluorescent analysis

GFP-LC3 and mCherry-DFCP1 puncta formation assays were performed as previously described [30, 31]. For GFP-LC3 puncta formation assay, HEK293 cells with GFP-LC3 stable expression were transfected with either DNA plasmids or siRNAs. For mCherry-DFCP1 puncta formation assay, different DNA plasmids was transfected into mCherry-DFCP1stably transfected HEK293 cells [11]. Before fixation, the GFP-LC3 cells were treated with 150 nM Baf A1 for 1 h and the mCherry-DFCP1 cells were treated with EBSS starvation for 2 h, respectively. Immunofluorescence staining of GULP1 and ATG14 was performed as previously described [19, 32, 33]. Images were captured with Leica TCS SP8 confocal microscope equipped with HC PL APO CS2 63x/1.40 OIL objective (Leica). The number of the fluorescent puncta is counted with ImageJ (NIH).

### Protein binding assays

GST fusion protein pull down and Coimmunoprecipitation assays were performed as described [20, 32]. In brief, GST-APP intracellular domain (AICD) fusion protein was expressed in E. coli BL21 and captured by Glutathione Sepharose 4B according to the manufacturer’s instructions (GE Healthcare). GST-GULP1 “baits” was used in pull-down assays from Flag-tagged ATG14 transfected cells which were harvested in ice-cold lysis buffer comprised of 50 mM Tris–HCl, pH 7.5, 150 mM NaCl, 1 mM EDTA, 1% Triton X100 and Complete^™^ protease inhibitor (Roche). The cell lysates were incubated with the baits at 4 °C for an hour. The captured proteins were then isolated by boiling in SDS-PAGE sample buffer and analysed by SDS-PAGE and immunoblotting. ATG14 was immunoblotted with M2 anti-FLAG antibody (Roche) against the C-terminal FLAG tag.

For immunoprecipitation, HEK293 cells transfected with ATG14-FLAG and HA-GULP1 were harvested in ice-cold lysis buffer. ATG14-FLAG was immunoprecipitated from cell lysates using mouse anti-FLAG antibody M2 (Sigma) for 16 h at 4 °C. The antibody was captured by protein G sepharose (Sigma) for 2 h at 4 °C and the immunoprecipitates were washed three times with ice-cold lysis buffer. Proteins in the immunoprecipitates were analyzed by SDS-PAGE and Western blotting.

For in vitro binding assay, GST or GST-GULP1 purified from *E. coli* were mixed with Ni–NTA resins coated with His6-ATG14 for 16 h at 4 °C. GST proteins in the pull-downs were detected by a rat anti-GST antibody.

### Proximity ligation assay

Proximity ligation assay (PLA) was performed by using a Duolink In Situ–Fluorescence kit (Sigma). In brief, HEK 293 cells were seeded on cover slips 24 h before transfection [20, 32, 33]. Cells were transfected with either GULP1, ATG14-FLAG or GULP1 + ATG14-FLAG. The cells were fixed with 4% paraformaldehyde and permeabilized with 0.1% Triton 24 h post transfection. After blocking with 5% FBS in PBS at 37 °C for 1 h, the cells were then incubated with goat anti-GULP1 P19 (Santa Cruz) and mouse anti-FLAG M2 (Sigma) for 1 h at room temperature to probe for GULP1 and ATG14, respectively. The cells were then washed three times with 1X Wash buffer A, followed by incubation with Duolink In Situ PLA probe anti-mouse PLUS and anti-goat MINUS at 37 °C for 1 h in a humid incubator. After incubation, the cells were washed three times with 1X Wash buffer A. Ligation was performed by adding 1X ligation stock and diluted ligase at 37 °C for 30 min, and then followed by two washes with 1X Wash buffer A. Amplification was carried out in a darkened humid incubator by incubating the cells with 1X amplification stock and diluted polymerase at 37 °C for 100 min. Then the cells were washed two times with 1X Wash buffer B and then one time with 0.01X Wash buffer B. The cover slips were mounted with Duolink In Situ mounting medium with DAPI. Images were captured by using a Nikon ECLIPSE Ni-U Upright Microscope. Fluorescence images were captured by a Nikon DS-Qi2 camera, and the fluorescence signals were quantified by the Object Count tool in Nikon NIS Elements. Cells were also stained with anti-β-tubulin as morphology marker.

### Isolation of endoplasmic reticulum

Endoplasmic reticulum (ER) was isolated as described [34]. In brief, HEK293 cells were trypsinized, washed with PBS and collected by centrifugation at 1000 g for 5 min. The cells were then suspended in 3X pelleted cell volume of ice-cold hypotonic extraction buffer (250 mM KCl, 10 mM EGTA, 100 mM HEPES, pH 7.8). After 20 min of incubation in the buffer at 4 °C, the cells were centrifuged at 1000 g, for 5 min at 4 °C. The cells are then suspended in a 2X pellet volume of ice-cold isotonic extraction buffer (125 mM KCl, 5 mM EGTA, 1.25 M sucrose, 50 mM HEPES, pH 7.8) and homogenized 10 strokes in a Dounce homogenizer. The homogenate was centrifuged at 1000 g for 10 min at 4 °C and the post-nuclear supernatant (PNS) was collected. PNS was further spun at 12,000 g for 15 min at 4 °C to obtain the post-mitochondrial supernatant (PMS). The PMS was transferred to a new vial and 7.5× volume of 8 mM CaCl2 was added in a dropwise manner with continuous mixing. After stirring for 15 min, the mixture was centrifuged at 8000 g, for 10 min at 4 °C. The ER-enriched pellet was resuspended in 1X SDS sample buffer and the amounts of ATG14, APP and GULP1 in the pellet were determined by Western blot analysis. The purity of the sample was analyzed by probing with antibodies against Calnexin and GAPDH.

### Isolation of the GFP-LC3-positive autophagosomal compartment

GFP-LC3-positive subcellular structure was isolated as described [35]. In brief, HEK293 cells were transfected with GFP-LC3 and indicated plasmids. The cells were resuspended in Buffer B (250 mM sucrose, 1 mM EDTA, 20 mM HEPES, pH 7.4) and lysed by passing through 22-gauge needles. Cell lysates were then centrifuged at 800 g for 10 min. The PNS was then centrifuged at 10,000 g for 20 min. The pellets were washed twice in the washing buffer (PBS, pH 7.4, 0.1% bovine serum albumin, 2 mM EDTA) to remove residual cytosolic GFP-LC3. The pellets were then resuspended in PBS with 2 mM EDTA and 3% bovine serum albumin, and the GFP-LC3-positive subcellular structure was isolated by immunoprecipitation using an anti-GFP antibody (JL-8). The samples were analyzed by immunoblotting.

### PI3KC3-C1 kinase assay

PI3KC3-C1 activity assay will be performed as described [36]. EV, wildtype GULP1, GULP1m cells were transfected ATG14, Beclin1, Vps 34 and Vps15. PI3KC3-C1 was immunoprecipitated from the transfected cell lysates by using an anti-ATG14 antibody. The immunoprecipitates were resuspended and incubated in 1 × substrate buffer containing 250 mg/ml phosphatidylinositol (PI) on ice for 10 min and the PIK3C3 kinase reactions were started by incubating 10 mM ATP for 30 min at room temperature. All the kinase reactions were spotted onto nitrocellulose membrane. The membrane will be probed with GST-p40-PX, a PI3P-specific lipid binding protein. The amount of GST-p40-PX bound to the reactions spotted on the membrane were determined by Western blot analysis using an anti-GST antibody.

### APP-GAL4 cleavage reporter assay

APP-GAL4 cleavage reporter assay was performed as described previously [19, 20]. Cells were transfected with the relevant constructs together with pRc-CMV-APP695, pFR-Luc and phRL-TK. phRL-TK which expresses the Renilla luciferase was used as a control to quantify transfection efficiency. The cells were harvested in Dual-Glo luciferase substrate (Promega) at 24 h post transfection. The firefly luciferase activities produced by pFR-Luc were measured by a luminometer (Perkin Elmer). Then, the Renilla luciferase activities produced by the phRL-TK were assayed by adding equal volume of Dual-Glo Stop&Glo substrate and analysed by the luminometer. The firefly luciferase activity was normalized to the corresponding Renilla luciferase activity.

### Tricine-SDS PAGE analysis for APP carboxyl-terminal fragments

APP carboxyl-terminal fragments (CTFs) from cells were separated by 16% Tricine-SDS PAGE. Immunoblot analyses were performed using a rabbit anti-APP antibody that recognizes the last 21 amino acid residues of APP [19, 20].

### Aβ ELISA assay

Human Aβ1-40 and Aβ1-42 levels in the cell culture medium were analyzed using the high sensitivity human amyloid β40 and amyloid β42 ELISA kits (Millipore) as described previously [19, 20]. In brief, HEK293 cells were transfected with human APP and indicated plasmids or siRNA. The cells were replenished with fresh medium 48 h post-transfection. After 7 h, the medium was collected, diluted as appropriate in sample diluent and added to the ELISA plate. After overnight incubation at the 4 °C with primary antibody, the ELISA plate was washed five times with wash buffer and streptavidin-peroxidase-conjugate was added. Following an one-hour room temperature incubation and washing, colorimetric substrate solution was added to the ELISA plate. The colorimetric signal development was then stopped by adding stop solution. Signals from ELISA were measured at 450 nm using a CLARIOstar microplate reader (BMG Labtech).

All experiments were repeated at least three times. Statistical analyses were performed using one-way ANOVA with Bonferroni post hoc test or unpaired t-test. Significance is indicated as ***p < 0.001; **p < 0.01; *p < 0.05; n.s., not significant (p > 0.05), respectively. Error bars show either S.D. or SEM.

## Results

### GULP1 potentiates autophagic flux

**Fig. 1:**
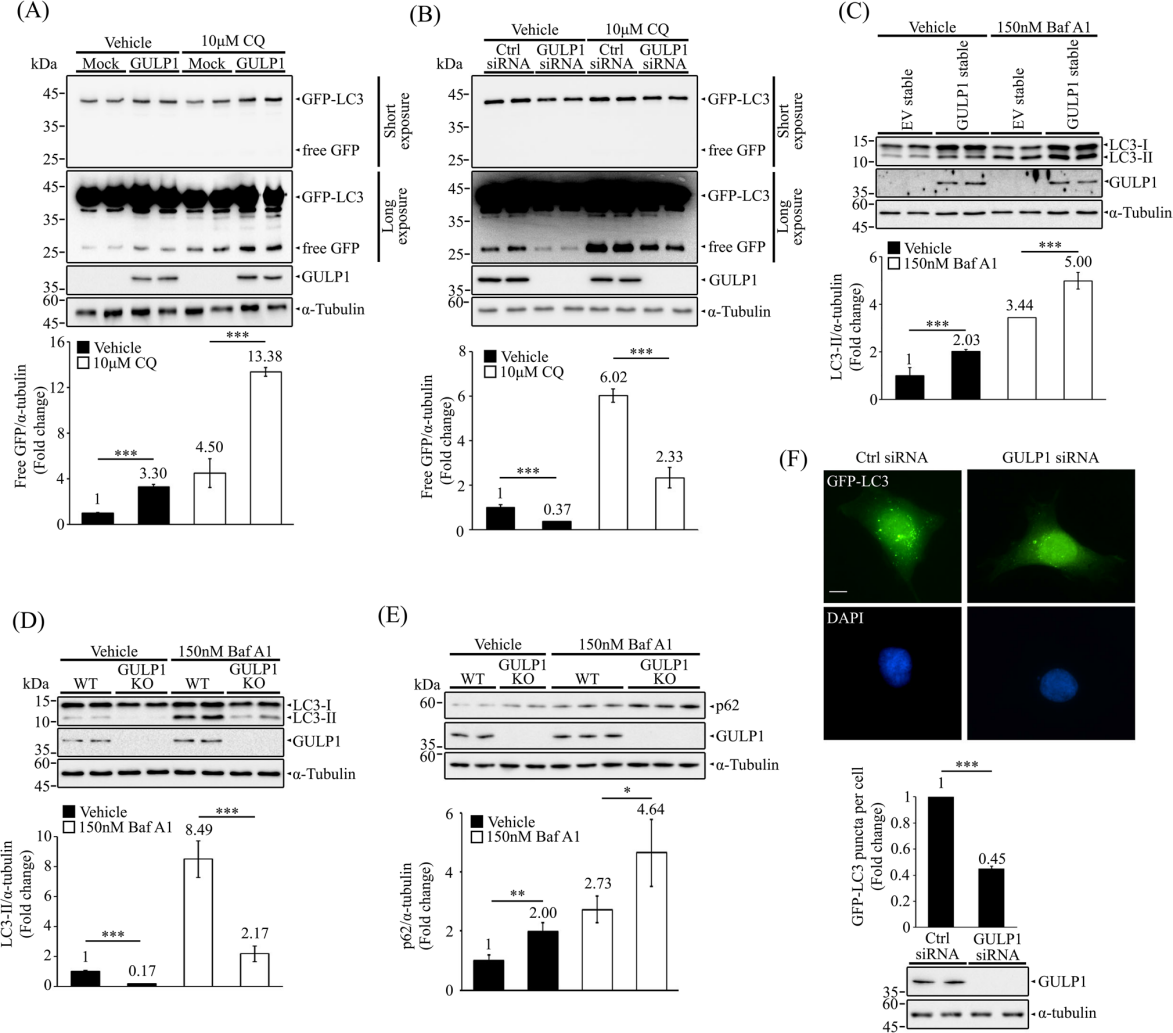
GULP1 potentiates autophagic flux. **A** Representative immunoblots for free GFP in HEK293 cells transfected with GFP-LC3 + mock or GFP-LC3 + GULP1 and treated as indicated. GFP proteins and GULP1 were detected with anti-GFP JL-8 and anti-GULP1 G-R3 respectively. α-tubulin was detected with anti-tubulin DM1A and used as a loading control. Bottom: quantification of relative GFP levels against loading control α-tubulin. Bar chart shows the densitometric quantification of relative GFP levels against α-tubulin. ***p < 0.001. **B** Immunoblotting of free GFP in GFP-LC3 transfected HEK293 cells with control or GULP1 siRNA KD treated as indicated. GFP proteins and GULP1 were detected with anti-GFP JL-8 and anti-GULP1 G-R3 respectively. α-tubulin was detected with anti-tubulin DM1A and used as a loading control. Bottom: quantification of relative GFP level against loading control α-tubulin. Bar chart shows the densitometric quantification of relative GFP levels against α-tubulin. ***p < 0.001. **C** Representative immunoblots for LC3 from EV and GULP1 stable transfected HEK293 cell lysates treated as indicated. LC3 and GULP1 were detected with anti-LC3 14600-1-AP and an anti-GULP1 G-R3 respectively. α-tubulin was detected with anti-tubulin DM1A and used as a loading control. Bar chart shows the densitometric quantification of relative LC3-II levels against loading control α-tubulin. ***p < 0.001. **D** Immunoblotting of LC3 from wild-type and GULP1-KO HEK293 cell lysates treated as indicated. LC3 and GULP1 were detected with anti-LC3 14600-1-AP and anti-GULP1 G-R3 respectively. α-tubulin was detected with anti-tubulin DM1A and used as a loading control. Bar chart shows the densitometric quantification of relative LC3-II levels against loading control α-tubulin. ***p < 0.001. **E** Immunoblot analysis of p62 in wild-type (WT) and GULP1-KO HEK293 treated as indicated. LC3 and GULP1 were detected with anti-LC3 14600-1-AP and an anti-GULP1 G-R3 respectively. α-tubulin was detected with anti-tubulin DM1A and used as a loading control. Bar chart shows the densitometric of relative p62 levels against α-tubulin. ***p < 0.001. **F** Top: representative images for GFP-positive puncta in control and GULP1 siRNA transfected HEK293 cells. Nuclei were stained with DAPI. Bar chart shows the quantification of GFP-positive puncta plotted by different siRNA transfection. Right bottom: immunoblot for GULP1 level in total cell lysates after siRNA transfection probed with anti-GULP1 G-R3. Data was obtained from at least 40 cells per transfection, and the experiment was repeated three times. Error bars are SEM. ***p < 0.001. Scale bar, 10 μm

To determine if GULP1 influences autophagy, GFP- microtubule-associated protein 1A/1B-light chain 3 (GFP-LC3) cleavage assay was employed. During autophagy, LC3 is degraded in autolysosomes. Hence, GFP-LC3 is recruited to autophagosomes after lipidation. As GFP is somewhat resistant to autolysosomal degradation, free GFP generated by the degradation of GFP-LC3 within autolysosomes can be used as a measure of autophagic flux [28]. We found that the release of GFP via GFP–LC3 degradation was markedly enhanced in GULP1-transfected cells and that these cells exhibited greater GFP accumulation relative to control cells upon treatment with chloroquine (CQ), a lysosomal proteinase inhibitor, which was used to exclude the possibility of impaired lysosomal degradation [29] (Fig. 1A). Conversely, the release of free GFP was significantly decreased in GULP1-knockdown (KD) cells compared with control cells, irrespective of CQ treatment (Fig. 1B). Moreover, increased endogenous levels of LC3-II, a commonly used marker for monitoring autophagy, were observed in cells stably transfected with GULP1 compared with control cells, regardless of treatment with bafilomycin A1 (BafA1), an inhibitor that prevents the fusion of autophagosomes and lysosomes and thus lysosomal degradation (Fig. 1C). Conversely, we observed decreased levels of LC3-II in GULP1-KO cells, irrespective of BafA1 treatment (Fig. 1D). We also monitored the level of p62, which accumulates when autophagy is inhibited, in GULP1-KO cells. Immunoblotting analyses showed that the levels of p62 were increased in GULP1-KO cells compared with control cells, with or without BafA1 treatment (Fig. 1E). Furthermore, KD of GULP1 in GFP–LC3-expressing HEK293 cells significantly reduced the number of GFP–LC3 puncta compared with the number observed in control cells (Fig. 1F). Taken together, our data suggest that GULP1 plays a role in autophagy.

### GULP1 interacts with ATG14

**Fig. 2:**
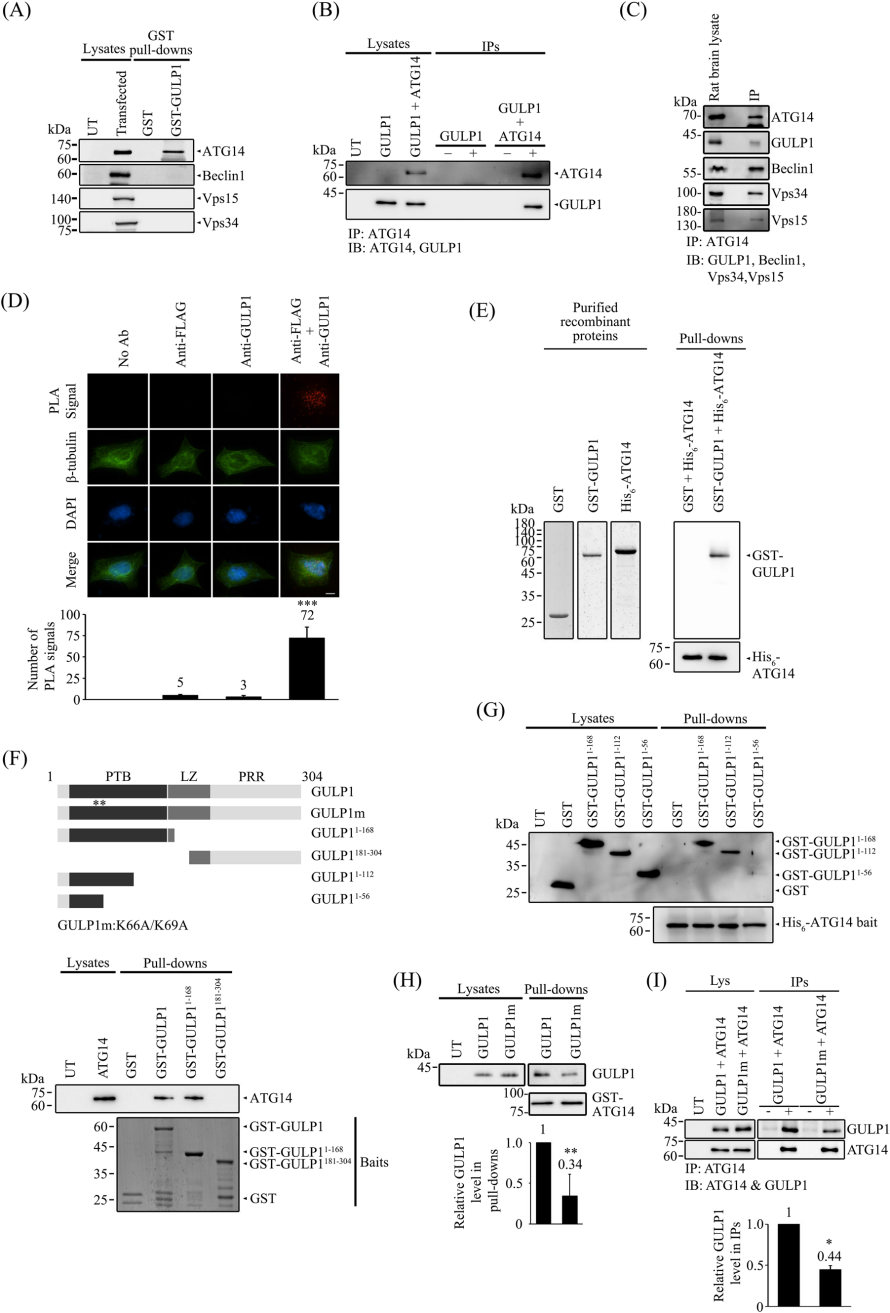
GULP1 interacts with ATG14. **A** Bacterially expressed GST and GST-GULP1 were used as baits for pull-down assay from ATG14, Beclin-1, VPS15 and VPS34 transfected cell lysate respectively. Overexpressed proteins in lysate and pull-downs were analyzed by immunoblotting. **B** Coimmunoprecipitation was performed from HEK293 cells transfected with ATG14 or ATG14 + GULP1. ATG14 in cell lysate was immunoprecipitated using a mouse anti-FLAG antibody. GULP1 and ATG14 in lysate and immunoprecipitates were analyzed by immunoblotting with anti-GUP1 G-R3 and anti-FLAG 20543-1-AP. **C** ATG14 in rat brain lysate was immunoprecipitated by an anti-ATG14 antibody. ATG14, GULP1, Beclin1, Vps34 and Vps15 in lysate and immunoprecipitate were immunoblotted with anti-ATG14 PD026, anti-GULP1 G-R3, anti-Beclin1 Bec-R3, anti-Vps34 F-11and anti-Vps15 JK-13. **D** In a fluorescent PLA assay, PLA signals representing GULP1-ATG14 interaction were detected in GULP1 + ATG14 transfected cells. Representative images are shown. Data were obtained from at least 60 cells per transfection and the experiments were repeated 3 times. Error bars are sem. ***p < 0.001. No-antibody (No Ab), anti-FLAG, and anti-GULP1 control PLAs were performed. **E** Bacterially expressed His_6_-ATG14 was used as baits to pull down purified GST or GST-GULP1. GST proteins were probed with a rat anti-GST serum. Left panel shows the purified protein used for pull-down. **F** Bacterially expressed GST and GST-GULP1 fragments were used as baits for pull-down assay from ATG14 transfected cell lysate. ATG14 in lysate and immunoprecipitates were immunoblotted with anti-ATG14 19,491-1-AP. Bottom panel: Coomassie Blue staining of GST-baits used. **G** Bacterially expressed His_6_-ATG14 was used as baits for pull-down assay from GST and GST-GULP1 fragments transfected cell lysate. GST proteins in lysate and immunoprecipitates were analyzed by immunoblotting. Lower panel: Coomassie Blue staining of His_6_-ATG14 baits. **H** Cells were co-transfected with GULP1 or GULP1 K66A/K69A (GULP1m). GST-fused ATG14 fragment was used as bait to pull down GULP1 in transfected cell lysates. GST-fusion proteins and GULP1 in lysates and pull-downs were probed with rat anti-GST serum and anti-GULP1 G-R3 respectively. Bar chart shows the densitometric quantification of relative GULP1 levels in pulldowns. **p < 0.01. **I** Cells were co-transfected with ATG14 + GULP1 or ATG14 + GULP1 K66A/K69A (GULP1m). ATG14 in cell lysate was immunoprecipitated using anti-FLAG M2 antibody. GULP1 and ATG14 in lysate and immunoprecipitates were immunoblotted with rat anti-GULP1 serum and anti-FLAG 20543-1-AP respectively. Bar chart shows the densitometric quantification of relative GULP1 levels in IPs. ***p < 0.001

Autophagy is a highly regulated process that involves the formation of distinct autophagic complexes at different stages. As GULP1 is a cellular adaptor protein with several protein–protein binding domains/regions, we speculated that GULP1 may participate in autophagy by binding to an autophagic complex. We found that BafA1, which blocks autophagosome–lysosome fusion, inhibited GULP1-mediated autophagy (Fig. 1C–E). Therefore, we focused on the complexes upstream of this fusion. PI3KC3-C1, which consists of Vps15, Vps34, ATG14 and Beclin1, is of interest as this complex is crucial for the initiation of autophagy [37, 38]. To determine whether GULP1 interacts with the components of PI3KC3-C1, cells were transfected with either ATG14, Beclin1, Vps15 or Vps34. GST-tagged GULP1 was the used to pull down the components separately from the corresponding transfected cell lysates. As shown in Fig. 2A, GST–GULP1 pulled down ATG14, but not the other PI3KC3-C1 components.

To confirm the interaction between GULP1 and ATG14, GULP1 was transfected into HEK293 cells either alone or with Flag-tagged ATG14. ATG14 was then immunoprecipitated from the cell lysates. GULP1 was detected in the immunoprecipitates of cells transfected with GULP1 + ATG14, but not in cells transfected with GULP1 only (Fig. 2B). The GULP1–ATG14 interaction was also detected in an immunoprecipitation assay of rat brain lysates (Fig. 2C), suggesting that the two proteins interact endogenously. Additionally, the other PI3KC3-C1 components including Beclin1, Vps15 and Vps34 were detected in the same immunoprecipitant (Fig. 2C), indicating that GULP1 interacts with PI3KC3-C1 by binding to ATG14. We further validated whether GULP1 and ATG14 interact in cells using a proximity ligation assay (PLA). As shown in Fig. 2D, in situ PLA signals were observed in cells labeled with antibodies against GULP1 and ATG14, confirming the presence of GULP1–ATG14 complexes. To examine whether the two proteins interact directly, we incubated GST or GST–GULP1 purified from *E. coli* with His_6_–ATG14 baits also purified from E. coli. GST–GULP1, but not GST, could be pulled down by His_6_–ATG14 (Fig. 2E). This finding indicates that GULP1 directly interacts with ATG14.

Next, we investigated the region within GULP1 that is essential for the GULP1–ATG14 interaction. Several truncated GST–GULP1 fusion proteins were used to pull down ATG14 from transfected cells. We found that the N-terminal fragment GULP1^1−168^, which contains the entire PTB domain, was sufficient to pull down ATG14 (Fig. 2F). Further pull-down assays using His6–ATG14 as the bait revealed that GULP1 residues 56 to 112 were important for the interaction, as GST-GULP1^1−168^ and GST-GULP1^1−112^, but not GST-GULP1^1−56^, could be pulled down by the bait (Fig. 2G). Using alanine screening mutagenesis, we found that the lysine 66 (K66) and lysine 69 (K69) residues of GULP1 were critical for the GULP1–ATG14 interaction. As shown in Fig. 2H and I, the GULP1–ATG14 interaction was markedly reduced in both GST pull-down and co-immunoprecipitation assays after introducing the K66A/K69A double mutation (GULP1m).

### GULP1 potentiates ATG14-mediated autophagy

**Fig. 3:**
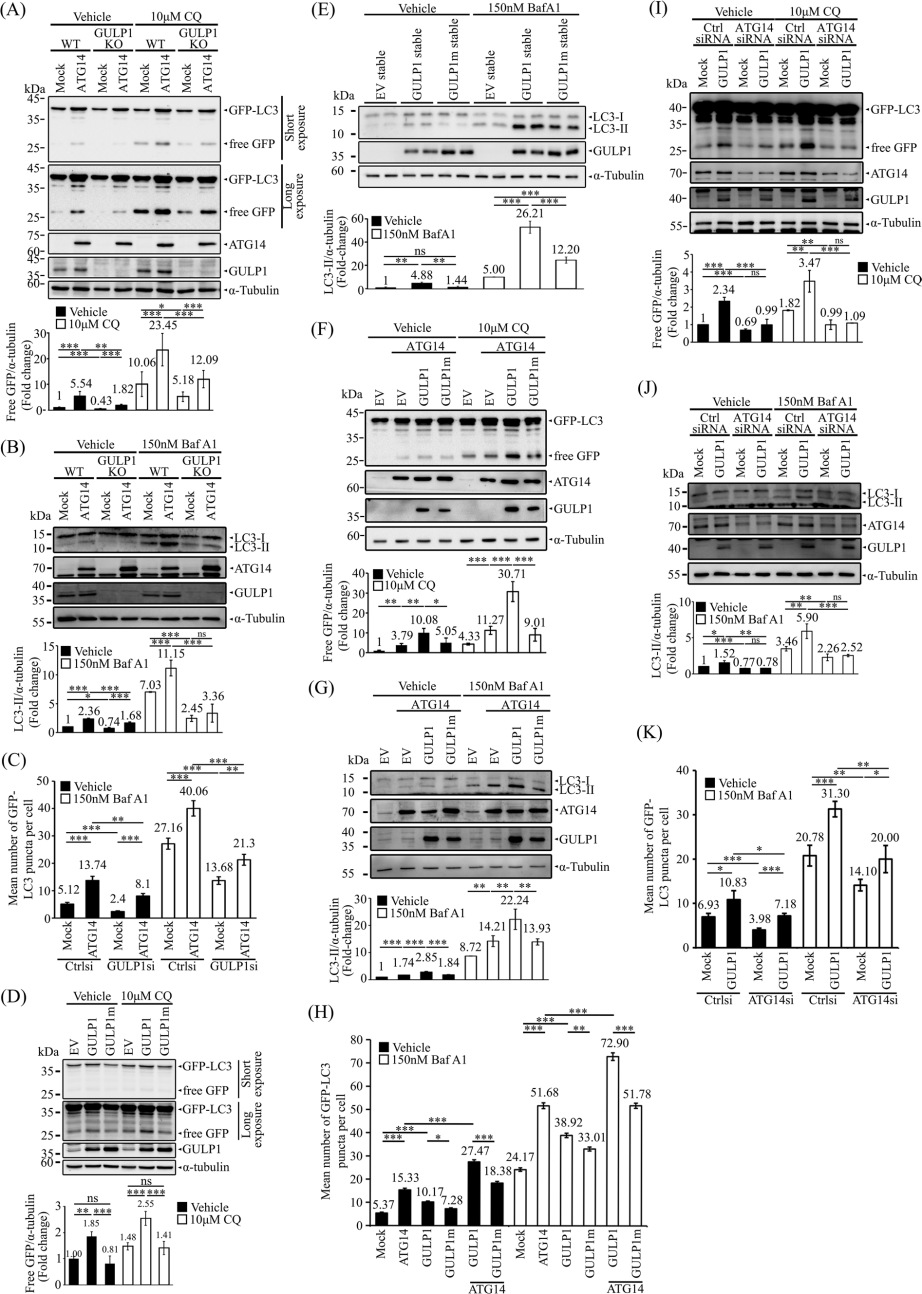
GULP1 potentiates ATG14-mediated autophagy. **A** Representative immunoblots for free GFP in WT and GULP1-KO HEK293 cells transfected with GFP-LC3 and treated as indicated. GFP fusion proteins, ATG14 and GULP1 were detected with anti-GFP JL-8, a mouse anti-FLAG antibody and anti-GULP1 G-R3 respectively. α-tubulin was detected with anti-tubulin DM1A and used as a loading control. Bottom: quantification of relative GFP levels against loading control α-tubulin. Bottom: densitometric quantification of relative GFP levels against α-tubulin. *p < 0.05, **p < 0.01, ***p < 0.001. **B** Representative immunoblots for LC3 from WT and GULP1 KO HEK293 cells transfected with ATG14 together with mock or ATG14. GULP1 were detected with anti-LC3 14600-1-AP, a mouse anti-FLAG antibody and an anti-GULP1 G-R3 respectively. α-tubulin was detected with anti-tubulin DM1A and used as a loading control. Bar chart shows the densitometric quantification of relative LC3-II levels against loading control α-tubulin. **p < 0.01, ***p < 0.001. *ns* not significant. **C** Quantification of GFP-LC3 puncta plotted by different transfection and treatment as indicated. Data was obtained from at least 40 cells per transfection, and the experiment was repeated three times. Error bars are sem. *p < 0.05, **p < 0.01, ***p < 0.001. **D** Representative immunoblots for free GFP in HEK293 cells stably transfected with EV, GULP1 and GULP1m and treated as indicated. GFP fusion proteins and GULP1 were detected with anti-GFP JL-8 and anti-GULP1 G-R3 respectively. α-tubulin was detected with anti-tubulin DM1A and used as a loading control. Bottom: quantification of relative GFP levels against loading control α-tubulin. Bottom: densitometric quantification of relative GFP levels against α-tubulin. *p < 0.05, **p < 0.01, ***p < 0.001. **E** Representative immunoblots for LC3 from EV, GULP1 and GULP1m stably transfected HEK293 cell lysates treated as indicated. LC3 and GULP1 were detected with anti-LC3 14600-1-AP and anti-GULP1 G-R3 respectively. α-tubulin was detected with anti-tubulin DM1A and used as a loading control. Bar chart shows the densitometric quantification of relative LC3-II levels against loading control α-tubulin. **p < 0.01, ***p < 0.001. *ns* not significant. **F** Immunoblotting of free GFP in HEK293 cells stably transfected with EV, GULP1 and GULP1m and transiently transfected with ATG14 and treatment as indicated. GFP fusion proteins, ATG14 and GULP1 were detected with anti-GFP JL-8, a mouse anti-FLAG antibody and an anti-GULP1 G-R3 respectively. α-tubulin was detected with anti-tubulin DM1A and used as a loading control. Bottom: quantification of relative GFP levels against loading control α-tubulin. Bottom: densitometric quantification of relative GFP levels against α-tubulin. *p < 0.05, **p < 0.01, ***p < 0.001. **G** Representative immunoblots for LC3 from HEK293 cells transfected with ATG14 together with EV, GULP1 or GULP1m. LC3, ATG14 and GULP1 were detected with anti-LC3 14600-1-AP, a mouse anti-FLAG antibody and an anti-GULP1 G-R3 respectively. α-tubulin was detected with anti-tubulin DM1A and used as a loading control. Bar chart shows the densitometric quantification of relative LC3-II levels against loading control α-tubulin. **p < 0.01, ***p < 0.001. **H** Quantification of GFP-LC3 puncta plotted by different transfection and treatment as indicated. Data was obtained from at least 40 cells per transfection, and the experiment was repeated three times. Error bars are sem. *p < 0.05, **p < 0.01, ***p < 0.001. **I** Representative immunoblots for free GFP in control- and GULP1-KD HEK293 cells transfected with GFP-LC3 together with mock or GULP1, and treated as indicated. GFP fusion proteins, ATG14 and GULP1 were detected with anti-GFP JL-8, a rabbit anti-ATG14 antibody and anti-GULP1 G-R3 respectively. α-tubulin was detected with anti-tubulin DM1A and used as a loading control. Bar chart shows densitometric quantification of relative GFP levels against α-tubulin. **p < 0.01, ***p < 0.001. **J** Representative immunoblots for LC3 from HEK293 cells transfected with control or ATG14 siRNA together with EV, GULP1 or GULP1m. LC3, ATG14 and GULP1 were detected with anti-LC3 14600-1-AP, a rabbit anti-ATG14 antibody and an anti-GULP1 G-R3 respectively. α-tubulin was detected with anti-tubulin DM1A and used as a loading control. Bar chart shows the densitometric quantification of relative LC3-II levels against loading control α-tubulin. **p < 0.01, ***p < 0.001. **K** Quantification of GFP-LC3 puncta plotted by different transfection and treatment as indicated. Data was obtained from at least 40 cells per transfection, and the experiment was repeated three times. Error bars are sem. *p < 0.05, **p < 0.01, ***p < 0.001

ATG14 is a crucial component of PI3KC3-C1 that stimulates autophagy [39, 40]. To investigate whether GULP1 potentiates autophagy through ATG14, GFP–LC3 cleavage assay and LC3-II analysis were performed. Overexpression of ATG14 potentiated the cleavage of GFP–LC3 and LC3-II level. The effect of ATG14 was significantly reduced in GULP1-KO cells, irrespective of BafA1 treatment (Fig. 3A and B). Likewise, KD of GULP1 reduced the number of GFP–LC3 puncta in in both vehicle- and BafA1-treated ATG14-overexpressing cells (Figs. 3C and S1A).

To determine the importance of the GULP1–ATG14 interaction, we used the binding defective GULP1m mutant. In both vehicle- and CQ-treated cells, the effect of GULP1m on GFP–LC3 cleavage was reduced significantly compared with the effect of wild-type GULP1 (Fig. 3D). Furthermore, increased levels of LC3-II were observed in cells stably expressing GULP1 but not in those stably expressing GULP1m, irrespective of BafA1 treatment (Fig. 3E). Moreover, both ATG14-mediated GFP–LC3 cleavage and LC3-II level were enhanced by GULP1 but not by the binding-defective mutant (Fig. 3F and G). Likewise, in both vehicle- and BafA1-treated cells, GULP1, but not GULP1m mutant, enhanced the formation of GFP–LC3 puncta mediated by ATG14 (Figs. 3H and S1B). Taken together, our data suggest that GULP1 interacts with ATG14 to influence autophagy.

To further investigate if the effect of GULP1 on autophagy is through ATG14, autophagy assays were performed in ATG14 KD cells. As shown in Fig. 3I, KD of ATG14 reduced GULP1-mediated GFP–LC3 cleavage, irrespective of BafA1 treatment. Similarly, GULP1 was unable to enhance LC3-II level in ATG14 KD cells (Fig. 3J). Additionally, the stimulatory effect of GULP1 on the formation of GFP–LC3 puncta decreased significantly in the KD cells (Figs. 3K and S1C). These observations further support the crucial role of ATG14 in mediating the effect of GULP1 on autophagy.

### GULP1 facilitates the ER targeting of ATG14 and the recruitment of APP to autophagic vacuoles

**Fig. 4:**
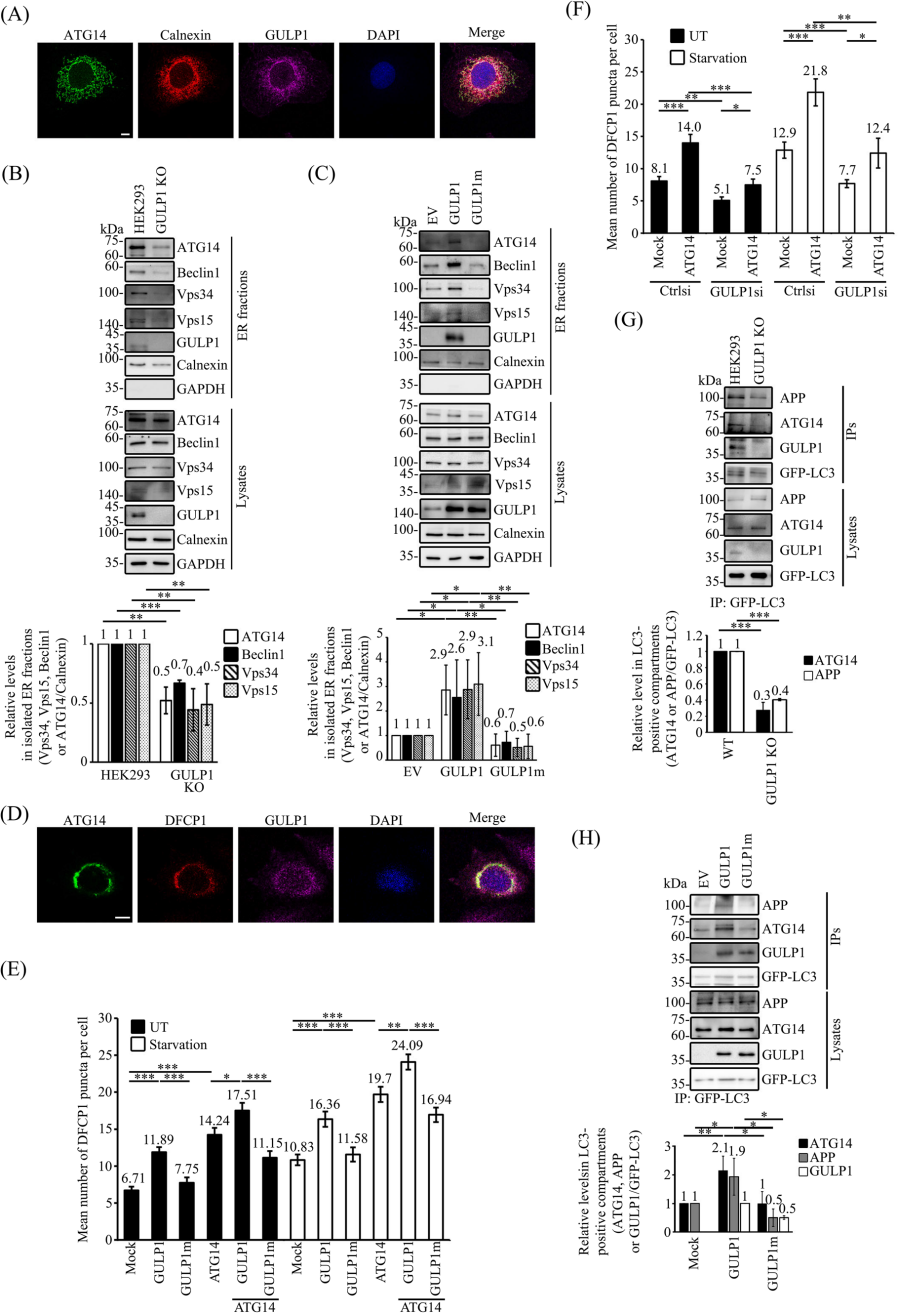
GULP1 facilitates the ER targeting of ATG14 and the recruitment of APP to autophagic vacuoles. **A** Immunostaining of COS7 cells for ATG14, calnexin and GULP1. ATG14, calnexin and GULP1 were stained by rATG14-2, 10427-2-AP and anti-GULP1 G-R3 respectively. An overlaid image is shown. Nuclei were stained with DAPI. Scale bar, 10 μm. **B** ER fractions were isolated from WT and GULP1-KO HEK293. Individual PI3KC3-C1 components in the isolated ER fractions were analyzed by immunoblotting by using anti-ATG14 PD026, anti-Beclin1 Bec-R3, anti-Vps34 F-11, anti-Vps15 JK-13 and anti-GULP1 G-R3, respectively. Subcellular compartment markers including calnexin and GAPDH were detected with anti-calnexin 10427-2-AP and anti-GAPDH AM4300 respectively. Data were obtained from three independent experiments. Bar chart shows the relative levels of PI3KC3-C1 components in GULP1-KO HEK293 cells compared to WT HEK293. **p < 0.01, ***p < 0.001. **C** ER fractions were isolated from EV, GULP1 and GULP1m stably transfected HEK293. Individual PI3KC3-C1 components in the isolated ER fractions were analyzed by immunoblotting by using anti-ATG14 PD026, anti-Beclin1 Bec-R3, anti-Vps34 F-11 and anti-Vps15 JK-13 respectively. Subcellular compartment markers including calnexin and GAPDH were detected with anti-calnexin 10427-2-AP and anti-GAPDH AM4300 respectively. Data were obtained from three independent experiments. Bar chart shows the levels of PI3KC3-C1 components relative to EV. *p < 0.05, **p < 0.01. **D** Immunostaining of CHO cells transfected with ATG14, mCherry-DFCP1 and GULP1. ATG14 and GULP1 were stained by rATG14-2 and an anti-GULP1 G-R3 respectively. An overlaid image is shown. Nuclei were stained with DAPI. Scale bar, 10 μm. **E** & **F** Quantification of mCherry-DFCP1-positive puncta plotted by different transfection and treatment as indicated. Data was obtained from at least 40 cells per transfection, and the experiment was repeated three times. Error bars are sem. *p < 0.05, **p < 0.01, ***p < 0.001. **G** WT and GULP1-KO HEK293 cells were transfected with GFP-LC3. Autophagic vacuoles were immunoprecipitated with anti-GFP JL-8 antibody. The protein content in total cell lysates and immunoisolated GFP-LC3 positive fractions was analyzed by anti-APP A5137, anti-ATG14 PD026 and anti-GULP1 G-R3. Bar chart shows the densitometric quantification of ATG14 and APP against GFP-LC3 in IPs. The experiment was repeated three times. ***p < 0.001. **H** Stable EV, GULP1 and GULP1m HEK293 cells were transiently transfected with GFP-LC3. Autophagic vacuoles were immunoprecipitated with anti-GFP JL-8 antibody. The protein content in total cell lysates and immunoisolated GFP-LC3 positive fractions was analyzed by anti-APP A5137, anti-ATG14 PD026 and anti-GULP1 G-R3. Bar chart shows the densitometric quantification of ATG14 and APP against GFP-LC3 in IPs. The experiment was repeated three times. *p < 0.05, **p < 0.01

ATG14 has been shown to localize to the endoplasmic reticulum (ER), a subcellular compartment involved in the biogenesis of autophagosomes [41]. We found that both endogenous ATG14 and GULP1 were colocalized with the ER marker calnexin (Fig. 4A). This observation suggests that GULP1 may influence ATG14-mediated autophagy, as well as APP processing, by influencing the targeting of ATG14 to the ER. To investigate this, we first fractionated ER from wild-type and GULP1-KO cells. Intriguingly, the amounts of ATG14, Beclin1, Vps34 and Vps15 in the ER fraction decreased significantly (Fig. 4B). Conversely, overexpression of GULP1, but not GULP1m, increased the amounts of ATG14 and the other PI3KC3-C1 components in the ER (Fig. 4C). These results suggest that the GULP–ATG14 interaction influences the formation of PI3KC3-C1.

Omegasomes are specialized cup-shaped membrane-bound compartments arisen from ER [42] which serve as progenitors of autophagosomes [42, 43]. Double FYVE-containing protein 1 (DFCP1) is an ER protein that has been implicated in the formation of omegasomes [44]. Our immunofluorescence analysis revealed that GULP1 was markedly colocalized with DFCP1 and ATG14 (Fig. 4D), which may suggest a role of GULP1 in the formation of omegasomes. To test this, we performed DFCP1-puncta-formation assays. As shown in Figs. 4E and S2A, GULP1, but not GULP1m, potentiated the formation of DFCP1-puncta in both mock-transfected and ATG14-co-transfected cells, irrespective of starvation treatment. In GULP1-KD cells, DFCP1-puncta formation was reduced significantly in both mock-transfected and ATG14-transfected cells (Figs. 4F and S2B).

We further isolated autophagic vacuoles (AVs) by immunoprecipitating transfected GFP–LC3 from cells. In GULP1-KO cells, the amount of ATG14 in the AVs (i.e. GFP–LC3 positive compartments) was reduced markedly (Fig. 4G). As APP is also highly localized in the ER and interacts with GULP1 [19, 45], we determined whether GULP1 alters the amount of APP in the AVs. Intriguingly, the amount of APP also decreased significantly in GULP1-KO cells (Fig. 4G). Conversely, the levels of ATG14 and APP increased significantly in cells stably transfected with GULP1, but not in those stably transfected with GULP1m (Fig. 4H). Our findings suggest that GULP1 promotes the targeting of ATG14 to the ER and simultaneously increases the localization of APP to the AVs.

### The GULP1-APP interaction enhances PI3KC3-C1 kinase activity

**Fig. 5:**
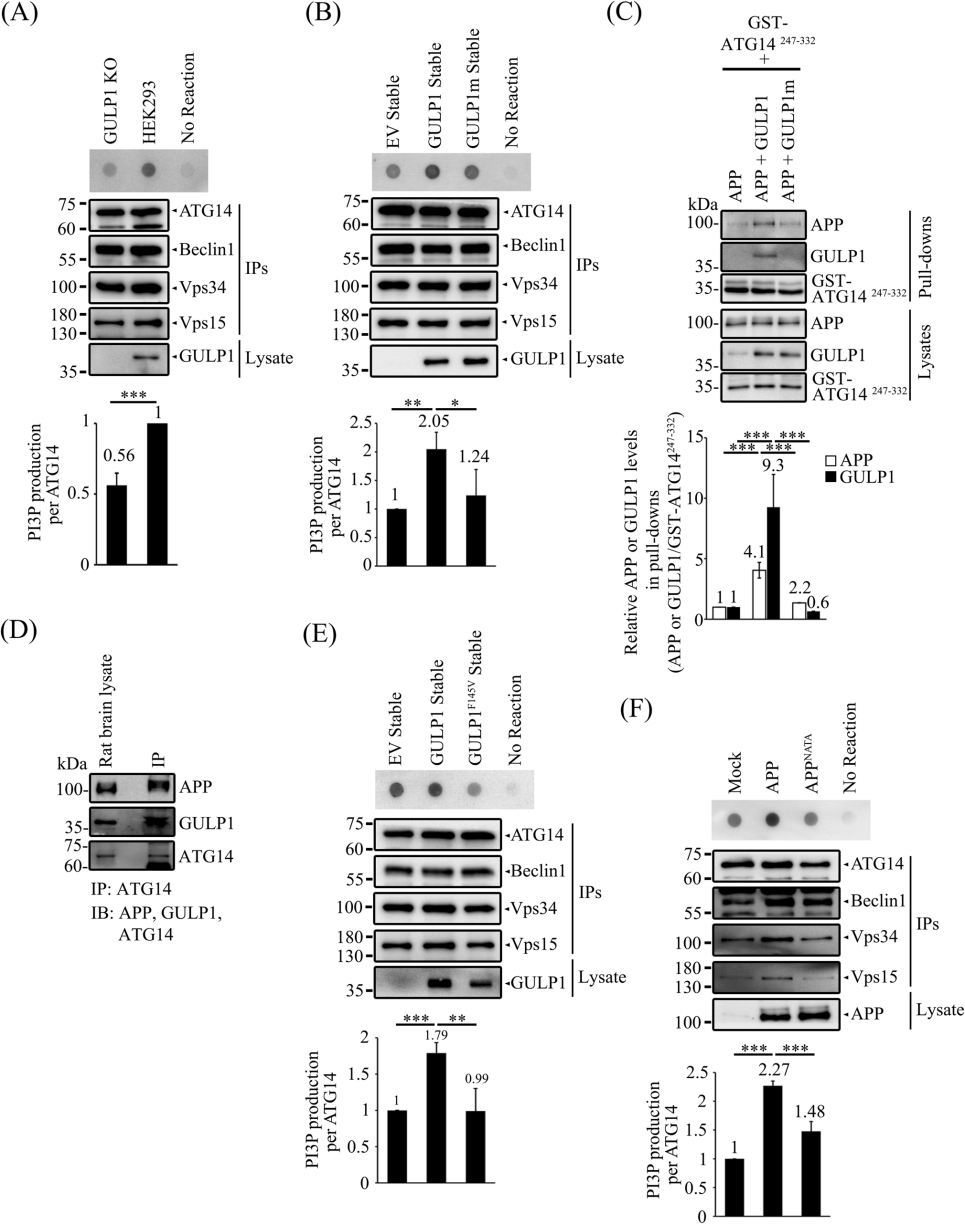
The GULP1-APP interaction enhances PI3KC3-C1 kinase activity. **A** WT and GULP1-KO HEK293 cells were transfected with ATG14, Beclin1, Vps34 and Vps15 and ATG14 was immunoprecipitated with anti-ATG14 anti-myc antibody 60003-2-IG. The immunoprecipitates were incubated with PI and ATP for 30 min. PI3P production was determined by dot blot and detected with GST-p40-phox. Bar chart shows the quantification of PI3P production normalized with immunoprecipitated ATG14. The experiment was repeated three times. ***p < 0.001. **B** Stable EV, GULP1 and GULP1m HEK293 cells were transfected with ATG14, Beclin1, Vps34 and Vps15 and ATG14 was immunoprecipitated with an anti-myc antibody 60003-2-IG. The immunoprecipitates were incubated with PI and ATP for 30 min. PI3P production was determined by dot blot and detected with GST-p40-phox. Bar chart shows the quantification of PI3P production normalized with immunoprecipitated ATG14. The experiment was repeated three times. ***p < 0.001. **C** CHO cells were transfected with GST-ATG14^247−332^ + APP, GST-ATG14^247−332^ + APP + GULP1 and GST-ATG14^247−332^ + APP + GULP1m. GST baits from cell lysates were captured by glutathione resins. Protein levels of APP, GULP1 and GST-ATG14^247−332^ were analysed with immunoblotting. Bar chart shows the densitometric quantification of co-precipitated APP and GULP1 relative to GST-ATG14^247−332^ baits. The experiment was repeated three times. ***p < 0.001. **D** ATG14 was immunoprecipitated with anti-ATG14 PD026 antibody from total rat brain lysate. APP, GULP1 and ATG14 in lysate and immunoprecipitates were analyzed by immunoblotting with anti-APP A5137, anti-GULP1 G-R3 and anti-ATG14 PD026. **E** Stable EV, GULP1 and GULP1^F145V^ HEK293 cells were transfected with ATG14, Beclin1, Vps34 and Vps15 and ATG14 was immunoprecipitated with anti-myc antibody 60003-2-IG. The immunoprecipitates were incubated with PI and ATP for 30 min. PI3P production was determined by dot blot. Bar chart shows the quantification of PI3P production normalized with immunoprecipitated ATG14. The experiment was repeated three times. ***p < 0.001. **F** HEK293 cells were transfected with ATG14, Beclin1, Vps34 and Vps15 and ATG14 and mock, APP or APP^NATA^ was immunoprecipitated with anti-myc antibody 60003-2-IG. The immunoprecipitates were incubated with PI and ATP for 30 min. PI3P production was determined by dot blot. Bar chart shows the quantification of PI3P production normalized with immunoprecipitated ATG14. The experiment was repeated three times. ***p < 0.001

PI3KC3-C1 is crucial for the initiation of autophagosome formation [22]. As GULP1 potentiates the formation of AVs via interaction with ATG14, we investigated whether GULP1 affects PI3KC3-C1 activity. PI3KC3-C1 was isolated from ATG14-, Beclin1-, Vps34- and Vps15-co-transfected cells by immunoprecipitating ATG14. We found that PI3P production decreased significantly in GULP1-KO cells (Fig. 5A). Conversely, PI3P production was augmented in cells stably transfected with GULP1, but not in those stably transfected with GULP1m (Fig. 5B). Taken together, our findings suggest that GULP1 influences AV formation by modulating PI3KC3-C1 activity via interaction with ATG14.

ER is subcellular compartment with high levels of APP [45]. APP may serve as a docking site of GULP1 for targeting ATG14 to the ER. We first investigated whether APP, GULP1 and ATG14 form a tripartite complex. Recombinant GST–ATG14^247−332^ fragment was used to pull down APP and GULP1 from the lysates of transfected cells. As shown in Fig. 5C, the amount of APP pulled down increased markedly in APP + GULP1 cell lysates compared with APP and APP + GULP1m cell lysates. In co-immunoprecipitation assays of rat brain lysates, both APP and GULP1 were co-immunoprecipitated with ATG14 (Fig. 5D).

Next, we investigated whether APP plays a role in autophagosome formation by modulating PI3KC3-C1 activity. To do this, PI3KC3-C1 activity was measured in cells transfected with APP and stably transfected with either GULP1 or the GULP1^F145V^ mutant, which has been shown to reduce the interaction with APP [19]. As shown in Fig. 5E, PI3KC3-C1 activity decreased significantly in cells transfected with GULP1^F145V^ compared with those transfected with wild-type GULP1. Likewise, overexpression of APP enhanced PI3KC3-C1 activity. However, this effect was not observed in cells transfected with the APP^NATA^ mutant, which does not bind GULP1 (Fig. 5F) [19]. These observations imply that the interaction between GULP1 and APP contributes, at least in part, to stimulating PI3KC3-C1 activity.

### ATG14 promotes GULP1-mediated APP processing

**Fig. 6:**
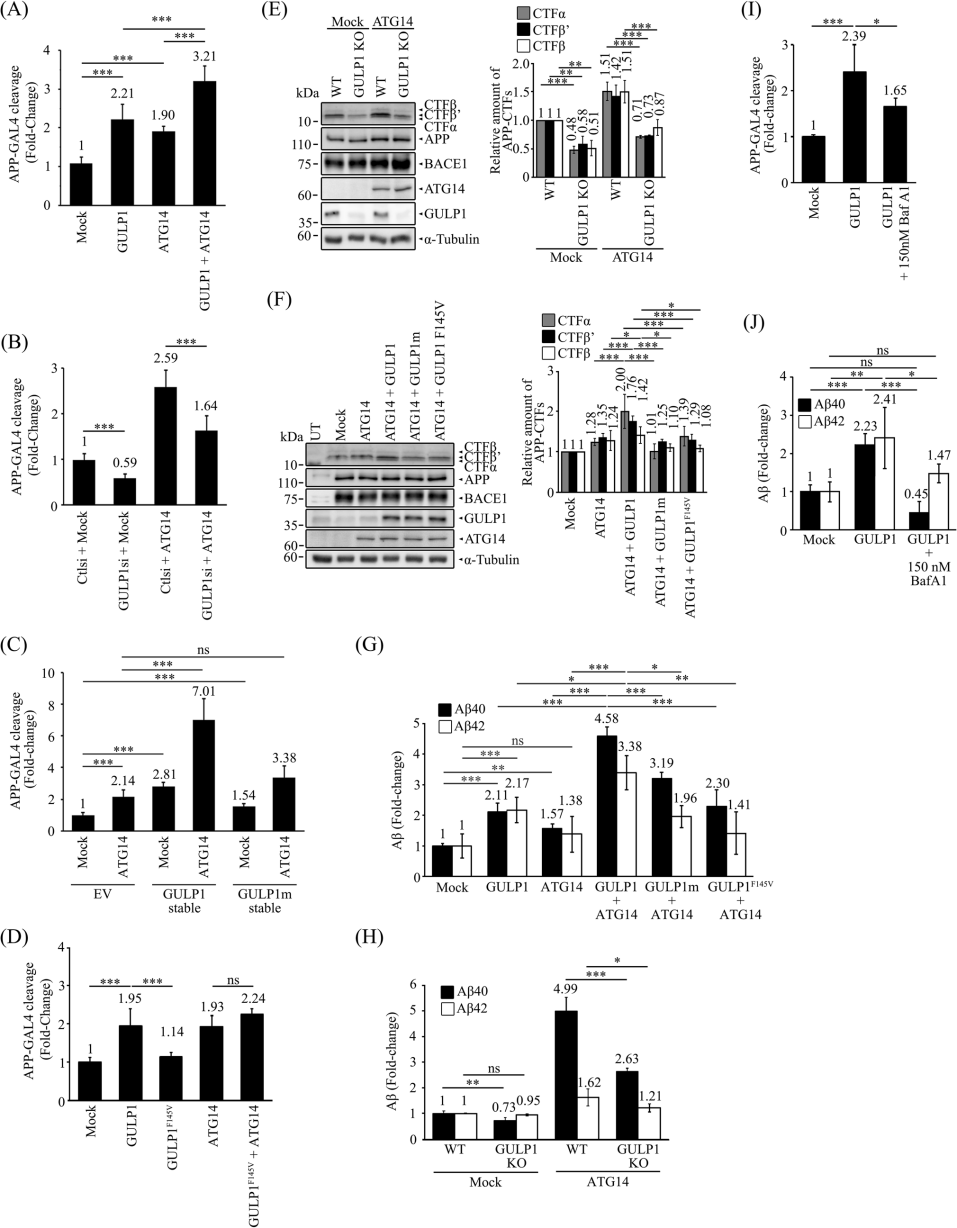
ATG14 promotes GULP1-mediated APP processing. **A**, **B** & **D** HEK293 cells were transfected with APP-GAL4, GAL4-reponsive firefly luciferase reporter, constitutive *Renilla* luciferase reporter and indicated expression constructs with either control or GULP1 siRNA. n = 5. Results are means ± SD. ***p < 0.001 compared with mock transfected cells. **C** HEK293 cell lines with indicated stable expression were transfected with APP-GAL4, GAL4-reponsive firefly luciferase reporter, constitutive *Renilla* luciferase reporter. n = 5. Results are means ± SD. ***p < 0.001 compared with mock transfected EV cells. *ns* not significant. Immunoblot analysis of APP CTFs from **E** wildtype and GULP1 KO HEK293 cells transfected with APP + BACE1 or APP + BACE1 + ATG14 **F** HEK293 cells transfected with APP + BACE1, APP + BACE1 + ATG14, APP + BACE1 + ATG14 + GULP1, APP + BACE1 + ATG14 + GULP1m or APP + BACE1 + ATG14 + GULP1^F145V^. The expression of transfected APP, ATG14, BACE1 and GULP1 in (**E**) and (**F**) were determined. Full-length APP and APP CTFs were detected by a rabbit anti-APP. HA-GULP1 and BACE1-myc were probed by a mouse anti-HA (12CA5) and a mouse anti-myc (9B11), respectively. The amounts of APP CTF-α, -β and -β’ were quantified. n = 3. **p < 0.01, ***p < 0.001; *ns* not significant. Results are means ± S.D. **G** HEK293 cells were cotransfected with APP and indicated expression plasmids. Forty-eight hours post-transfection, cell culture medium was aspirated and changed to fresh medium. The levels of secreted Aβx-40 and Aβx-42 were assayed using an ELISA kit 7 h after the change of medium. n = 5. *p < 0.05, **p < 0.01, ***p < 0.001 compared with mock transfected cells. **E** Wild-type and GULP1-KO HEK293 cells were cotransfected with APP and indicated expression plasmids. Aβx-40 and Aβx-42 were assayed as described in (**H**). n = 5. *p < 0.05, **p < 0.01, ***p < 0.001 compared with mock transfected cells. **I** APP GAL-4 cleavage **J** Aβ ELISA assays were performed in HEK293 cells transfected with mock, GULP1 and GULP1 treated with 150 nM BafA1 for 24 h. n = 5. *p < 0.05, **p < 0.01, ***p < 0.001. *ns* not significant

It has been suggested that autophagosomes are sites of Aβ generation [1, 46]. As we have shown here, GULP1 increases the amount of APP in AVs. Therefore, we investigated the effect of ATG14 on GULP1-mediated APP processing. We used an APP–GAL4 cleavage reporter assay to monitor the generation of the APP intracellular domain (AICD). In this assay, AICD–GAL4 is liberated by γ-secretase cleavage of recombinant APP-GAL4. AICD–GAL4 is then translocated to the nucleus to stimulate the transcription of the GAL4-dependent firefly luciferase reporter gene. As shown in Fig. 6A, overexpression of either GULP1 or ATG14 alone promoted APP–GAL4 cleavage. The cleavage was further enhanced in GULP1-and-ATG14-co-transfected cells. Conversely, siRNA KD of GULP1 significantly suppressed the effect of ATG14 on APP–GAL4 cleavage (Fig. 6B). Notably, ATG14 overexpression potentiated APP–GAL4 cleavage in cells stably transfected with GULP1, whereas this effect was inhibited in cells stably expressing GULP1m (Fig. 6C).

As shown in Figs. 5D and 6C, APP, GULP1 and ATG14 form a tripartite complex and the interaction of GULP1 and ATG14 is essential for APP processing. To evaluate the importance of APP-GULP1 interaction in GULP1-ATG14-mediated APP processing, GULP1^F145V^ mutant was employed. As shown in Fig. 6D, the effect of GULP1 on APP–GAL4 cleavage was markedly reduced by the GULP1^F145V^ mutation. Moreover, the mutant could not potentiate the effect of ATG14 on APP processing as the wildtype GULP1, as illustrated in Fig. 6A.

We also determined the effect of ATG14 and GULP1 on the production of APP C-terminal fragments (CTFs). Overexpression of ATG14 increased the production of APP CTF-α, -β and -β’ in wildtype cells but not the GULP1 KO cells (Fig. 6E). Moreover, only GULP1, but not the GULP1^F145V^ and GULP1m mutants, could potentiate ATG14-mediated APP CTF production (Fig. 6F). Thus, the formation of APP-GULP1-ATG14 complex is essential for GULP1-ATG14-mediated APP processing.

Next, we examined the effect of the GULP1–ATG14 interaction on Aβ secretion. As previously reported, GULP1 promotes both Aβ40 and Aβ42 secretion. Overexpression of ATG14 also stimulated Aβ40 and Aβ42 secretion (Fig. 6G). More pronounced Aβ secretion was observed in cells co-transfected with GULP1 and ATG14 (Fig. 6G). This synergetic effect of GULP1 and ATG14 on Aβ40 and Aβ42 secretion were markedly reduced in cells co-transfected with GULP1m and ATG14 (Fig. 6D). Likewise, GULP1^F145V^ mutant could not potentiate the effect of ATG14 on Aβ secretion (Fig. 6G). Additionally, the effect of ATG14 on Aβ40 and Aβ42 secretion were significantly reduced in GULP1-KO cells compared with control cells (Fig. 6H). Collectively, our data suggest that GULP1 and ATG14 function cooperatively to potentiate APP processing.

We further determined whether the effect of GULP1 on APP processing is through autophagy. Hence, we examined APP processing in cells treated with the autophagy inhibitor Baf A1. As shown in Fig. 6I and J, both APP–GAL4 cleavage and Aβ secretion were notably decreased in GULP1 transfected cells after treatment with Baf A1, indicating that GULP1, at least in part, regulates APP processing via autophagy.

## Discussion

Autophagy is a cellular process that is critical for maintaining cellular homeostasis by degrading and recycling damaged or unwanted cellular components. The dysregulation of autophagy has been implicated in the pathogenesis of neurodegenerative diseases, including AD. Mounting evidence suggests that the balance between the production and clearance of disease-associated protein species, such as Aβ in AD, by autophagy is crucial. PI3KC3-C1 is a key autophagic complex, as it plays a significant role in the formation of autophagosomes. ATG14 is a major molecule in the complex, and it is essential for regulating the formation and localization of PI3KC3-C1 [47]. Several studies have suggested that the molecular adaptor GULP1 plays a role in autophagy [12–15], but the precise mechanism has not yet been clarified. In the present study, we found that GULP1 enhances autophagy by interacting with ATG14. Moreover, this interaction potentiated PI3KC3-C1 activity. It is also known that PI3KC3-C1 activity can be regulated by phosphorylation [22]. Notably, GULP1 interacts with Jedi-1 to facilitate its phosphorylation to stimulate phagocytosis [48]. It is possible that GULP1 acts as an adaptor to recruit a kinase(s) for regulating PI3KC3-C1 activities via the phosphorylation of PI3KC3-C1 components. Nevertheless, our findings provide evidence that GULP1 acts as a regulator of autophagy via direct interaction with ATG14 to influence PI3KC3-C1 activity.

The ER plays a crucial role in the de novo synthesis of membrane-bound structures and organelles, including the establishment of an ER subdomain, namely the omegasome, which is an early step in the biogenesis of autophagosomes [49]. ATG14 has been reported to function in the recruitment of Vps34 to the ER, which is essential for membrane curvature sensing during omegasome formation [50, 51]. In this study, we found that GULP1 increases the level of ATG14 in the ER, which may facilitate the recruitment of Vps34 and other components of PI3KC3-C1 to the ER to trigger biogenesis of the omegasome. Consistently, GULP1 enhanced GFP-LC3 puncta formation, which reflects the number of autophagy-related structures in cells. The ER is also a compartment involved in the folding and maturation of APP [52]. Noteworthy, APP has been demonstrated to affect autophagic activity [53]. Our findings support this observation, as the overexpression of APP enhanced PI3KC3-C1 activity. Intriguingly, this effect was not observed in cells transfected with the APP^NATA^ mutant, suggesting that the NPXY motif within the AICD and/or its binding protein(s) are essential for APP-mediated PI3KC3-C1 activity. This notion was further supported by the observation that GULP1 enhanced PI3KC3-C1 activity, while the GULP1^F145V^ mutant, which is defective in binding to the AICD [19], did not have the same effect. As APP has been proposed to function as a membrane docking site for its interactor [54], it may serve as a docking site at the ER for the recruitment of GULP1 and/or the GULP1–ATG14 complex.

As mentioned, GULP1 also interacts with APP to alter Aβ production by a yet to be identified mechanism(s). Aβ is generated from the proteolytic cleavage of APP by β- and γ-secretases. Both of these secretases have been detected in autophagosomes [55, 56]. It has been proposed that autophagosomes are sites of Aβ production [1, 46]. In this study, ATG14 was shown to enhance GULP1-mediated APP processing and Aβ generation, indicating that GULP1 partly alters APP processing through autophagy. Of note, the level of APP in AVs was increased in the presence of GULP1, suggesting that GULP1 may play dual roles in autophagy-mediated APP processing by (i) enhancing the targeting of ATG14 to the ER and (ii) facilitating the entry of APP to AVs. Notably, we demonstrated that GULP1 facilitates both PI3KC3-C1 activity and Aβ secretion which is consistent with some reports suggesting a positive correlation between autophagy/PI3KC3-C1 activity and Aβ production. For example, curcumin has been shown to inhibit both PI3K expression and Aβ generation [57]. Additionally, autophagy and Aβ levels decreased in AD mice treated with β-asarone [58]. Conversely, some molecules have been reported to reduce Aβ production by enhancing PI3KC3-C1 activity such as NRBF2 [59, 60]. Furthermore, the precise effects of GULP1 on APP processing and Aβ production remain unclear [19–21, 25]. While the reasons for the above conflicting observations are not yet clear, some regulators of autophagy have been reported to have a differential effect on the process in different cell types. For instance, mammalian target of rapamycin complex 2 (mTORC2) acts as a suppressor of autophagy in skeletal muscle [61], while reactive oxygen species-induced mTORC2 activity enhances autophagy in fibroblasts [62, 63]. Additionally, glycogen synthase kinase-3β has been shown to increase autophagy in rat hippocampal neural stem cells [64] but to suppress the process in MCF-7 breast cancer cells and L6 rat skeletal muscle cells [65, 66]. Likewise, Rac1 has disparate effects on autophagy in different cell types [67–70]. These findings suggest that the same regulatory factor may modulate autophagy differently in different cell types [71]. Our data indicate that GULP1 influences APP processing, at least partially, through autophagy. The conflicting effect on GULP1-mediated APP processing may be attributed to variations in the expression of GULP1 and its interplay with other regulators of autophagy in different cell types.

APP is expressed in different tissues and is implicated in different cellular processes [72]. In addition to GULP1, the NPXY motif of the AICD binds to other cellular adaptors, and some of them are also implicated in autophagy. For example, the KD of Numb triggers the accumulation of AVs and reduces autophagic degradation in MCF-7 cells [73]. Increased expression levels of autophagic biomarkers have been observed in different regions of the embryonic kidney in Dab1-KO mice [74]. Moreover, JIP1 serves as a regulator for the transportation of autophagosome in neurons [75]. Notably, AICD interactors have been shown to compete for the binding to APP [76]. Although the precise connection between APP and these adaptors in autophagy is yet to be determined, it is possible that APP recruits different AICD-interacting adaptors, including GULP1, to the ER to fine-tune autophagic activity in different tissues and/or developmental stages.

Studies of *Atg*-gene-KO mice have revealed that Atg is essential for survival, as the *Atg*-KO animals showed either embryonic or neonatal lethality, implying that autophagy is crucial for development [77]. Furthermore, mutations in *Atg* genes and the dysregulation of autophagy-associated pathways have been associated with certain neurodevelopmental disorders, such as autism spectrum disorder [78]. The findings of recent studies have revealed the involvement of GULP1 in development. It has been demonstrated that GULP1 regulates Eph/ephrin-mediated trogocytosis during embryonic development [79]. Moreover, the absence of GULP1 leads to a reduction in the differentiation of osteoclasts [17]. Given that GULP1 is expressed in the embryonic neurons and brain [19], our finding that GULP1 interacts with ATG14 to modulate autophagy provides a new avenue for investigating the role(s) of GULP1 in neurodevelopment through its effects on autophagy.

Increasing evidence suggests an interplay between the endocrine system and autophagy [18, 80]. For instance, insulin is an anabolic hormone that has been found to suppress autophagy [81, 82]. Notably, insulin also inhibits Aβ production and plays a role in neuroprotection [83–86]. However, the exact mechanism by which insulin exerts such neuroprotective effects remains elusive. Previously, we demonstrated that GULP1-mediated APP processing is enhanced by atypical protein kinase C (aPKC)-mediated phosphorylation of GULP1, which inhibits GULP1-APP interaction [20]. Previous studies have shown that insulin can activate aPKC and inhibit autophagy [87–89]. As our finding suggests that APP acts as a docking site at the ER for GULP1, it is possible that insulin suppresses autophagy-mediated APP processing via activation of aPKC-mediated phosphorylation of GULP1, resulting in fewer GULP1-APP interaction and consequently diminishing the targeting of GULP1 or the GULP1–ATG14 complex to the ER.

Disrupting the dynamic balance of autophagy can affect the production and clearance of proteins, and this is closely linked to the development of neurodegenerative disorders [90]. While the direct targeting of ATGs is a straightforward approach to altering autophagy, it may pose significant undesirable effects, as ATGs are crucial for survival. Therefore, a greater understanding of the mechanisms that regulate autophagy will provide important insights into alternate methods and targets to modulate autophagy. Our findings that GULP1 is a novel regulator of autophagy and APP processing open another avenue for modifying the autophagic process to control the production and/or clearance of disease-related proteins.

### Supplementary Information

Below is the link to the electronic supplementary material.

Supplementary file1 Representative images for GFP- LC3-positive puncta in the cells for (A) Fig. 3B (B) 3F (C) 3K (PDF 525 KB)

Supplementary file2 Representative images for mCherry-DFCP1-positive puncta in the cells for (A) Fig. 4E (B) 4F (PDF 532 KB)

## Data Availability

This article has no additional data.

## Abbreviations

Aβ: Amyloid-β peptide
AD: Alzheimer’s disease
AICD: APP intracellular domain
aPKC: Atypical protein kinase C
APP: Amyloid precursor protein
ATP: Adenosine triphosphate
AVs: Autophagic vacuoles
ATG14: Autophagy-related 14
Baf A1: Bafilomycin A1
BSA: Bovine serum albumin
CED-6: *Caenorhabditis elegans* cell death protein 6
*C. elegans*: *Caenorhabditis elegans*
CHO: Chinese hamster ovary
CQ: Chloroquine
DFCP1: Double FYVE-containing protein 1
EBSS: Earle’s balanced salt solution
ELISA: Enzyme-linked immunoassay
ER: Endoplasmic reticulum
EV: Empty vector
FBS: Fetal bovine serum
GAPDH: Glyceraldehyde-3-phosphate dehydrogenase
GFP: Green fluorescent protein
GST: Glutathione-s-transferase
GULP1: Engulfment adaptor phosphotyrosine-binding domain-containing protein 1
HEK293: Human embryonic kidney 293
HEPES: 4-(2-Hydroxyethyl)-1-piperazineethanesulfonic acid
JIP-1: JNK-interacting protein 1
KD: Knock down
KO: Knock out
LC3: Microtubule-associated protein 1A/1B-light chain 3
MCF-7: Michigan cancer foundation-7
mTORC2: Mammalian target of rapamycin complex 2
PEI: Polyethylenimine
PI: Phosphatidylinositol
PICALM: Phosphatidylinositol binding clathrin assembly lymphoid-myeloid leukemia
PI3KC3-C1: Class III phosphatidylinositol 3-kinase complex 1
PI3P: Phosphatidylinositol 3-phosphate
PLA: Proximity ligation assay
PMS: Post-mitochondrial supernatant
PNS: Post-nuclear supernatant
PVDF: Polyvinylidene difluoride
Rac1: Ras-related C3 botulinum toxin substrate 1
WT: Wild type
