# Supplementary figures and images for: The cellular adaptor GULP1 interacts with ATG14 to potentiate autophagy and APP processing
Source: Cell Mol Life Sci. 2024 Jul 30;81(1):323. doi: 10.1007/s00018-024-05351-8 (PMC11335243; doi:10.1007/s00018-024-05351-8)

Figure S1

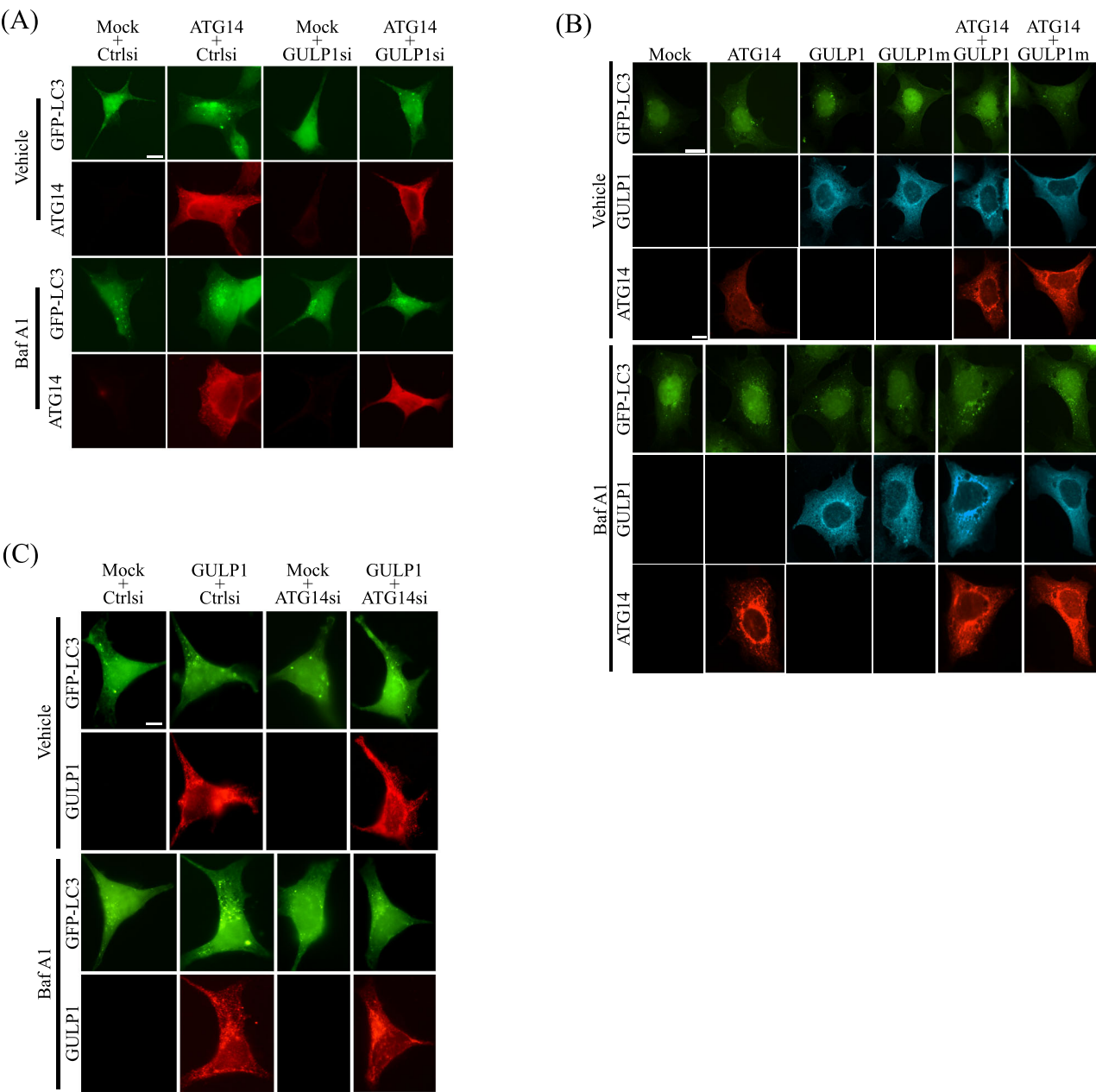

Supplement: Supplementary file 1 — Supplementary file1 Representative images for GFP- LC3-positive puncta in the cells for (A) Fig. 3B (B) 3F (C) 3K (PDF 525 KB) [file 18_2024_5351_MOESM1_ESM.pdf]

Figure S2

(A)

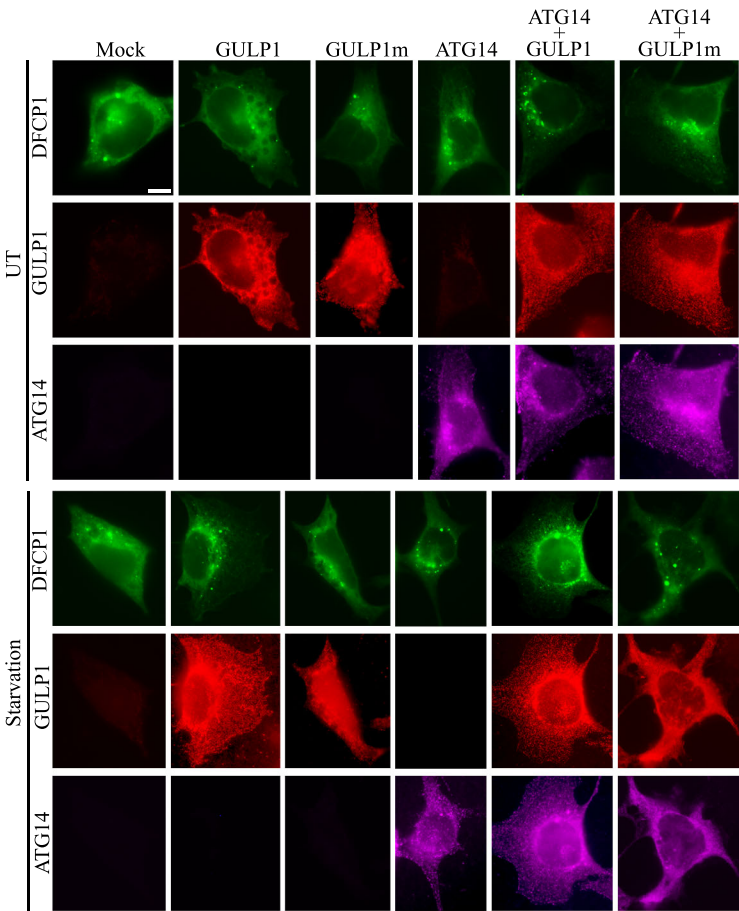

(B)

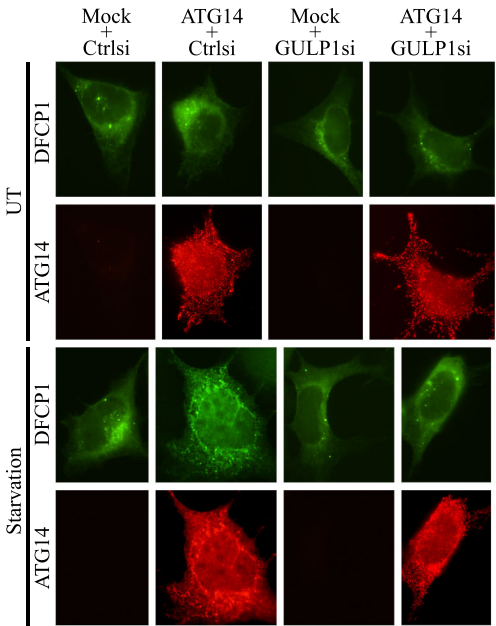

Supplement: Supplementary file 2 — Supplementary file2 Representative images for mCherry-DFCP1-positive puncta in the cells for (A) Fig. 4E (B) 4F (PDF 532 KB) [file 18_2024_5351_MOESM2_ESM.pdf]
